# Vascular Grafts: Technology Success/Technology Failure

**DOI:** 10.34133/bmef.0003

**Published:** 2023-01-16

**Authors:** Buddy Ratner

**Affiliations:** Center for Dialysis Innovation (CDI), Departments of Bioengineering and Chemical Engineering, University of Washington, Seattle, WA 98195, USA.

## Abstract

Vascular prostheses (grafts) are widely used for hemodialysis blood access, trauma repair, aneurism repair, and cardiovascular reconstruction. However, smaller-diameter (≤4 mm) grafts that would be valuable for many reconstructions have not been achieved to date, although hundreds of papers on small-diameter vascular grafts have been published. This perspective article presents a hypothesis that may open new research avenues for the development of small-diameter vascular grafts. A historical review of the vascular graft literature and specific types of vascular grafts is presented focusing on observations important to the hypothesis to be presented. Considerations in critically reviewing the vascular graft literature are discussed. The hypothesis that perhaps the “biocompatible biomaterials” comprising our vascular grafts—biomaterials that generate dense, nonvascularized collagenous capsules upon implantation—may not be all that biocompatible is presented. Examples of materials that heal with tissue reconstruction and vascularity, in contrast to the fibrotic encapsulation, are offered. Such prohealing materials may lead the way to a new generation of vascular grafts suitable for small-diameter reconstructions.

## Introduction

Vascular prostheses (grafts) are widely used for hemodialysis blood access, trauma repair, aneurism repair, and cardiovascular reconstruction. General requirements for success in all these applications are similar: The graft must not thrombose, the graft must not trigger hyperplasia, the graft must not lose mechanical integrity, and the graft must meet surgeon handling requirements (e.g., suturability).

Vascular grafts used in surgery today are composed primarily of expanded Teflon (ePTFE) or Dacron fabric. Newer approaches based on tissue engineering or reprocessed natural tissue are in clinical trials.

Entering the term “vascular graft” (in quotation marks) in the Google Scholar website gives 48,800 hits, evidence of the interest in these devices, their impact, and the degree to which they have been studied. Yet, vascular grafts have been unable to meet certain imperative needs in medicine. There are no regulatory agency-approved small-diameter vascular grafts (≤4 mm inner diameter). There is no widely accepted definition of a small-diameter vascular graft. Many articles propose <6 mm. In this article, I use ≤4 mm. Such grafts could be used for limb blood vessel replacement, possibly saving many of the roughly 1 million limbs amputated each year worldwide. Even smaller-diameter grafts could simplify cardiac bypass procedures (approximately 400,000 cardiac bypass procedures performed in the United States each year). Arterio-venous grafts used for dialysis access also have a high failure rate at 1 to 2 years, leading to expensive reoperations. Grafts fail primarily due to thrombosis and hyperplasia (excess cell proliferation). These factors are interrelated in that hyperplastic narrowing of vessel lumen slows blood flow and increases the probability of thrombosis. In the 70+ years since the first synthetic vascular grafts were developed, these failure modes still plague the medical community. With so many published studies, one might imagine that solutions to less-than-ideal graft performance might have been realized and introduced to the clinic, but this has not happened. This highlights the complexity of the engineering–biology–medicine problem.

**Fig. 1.**
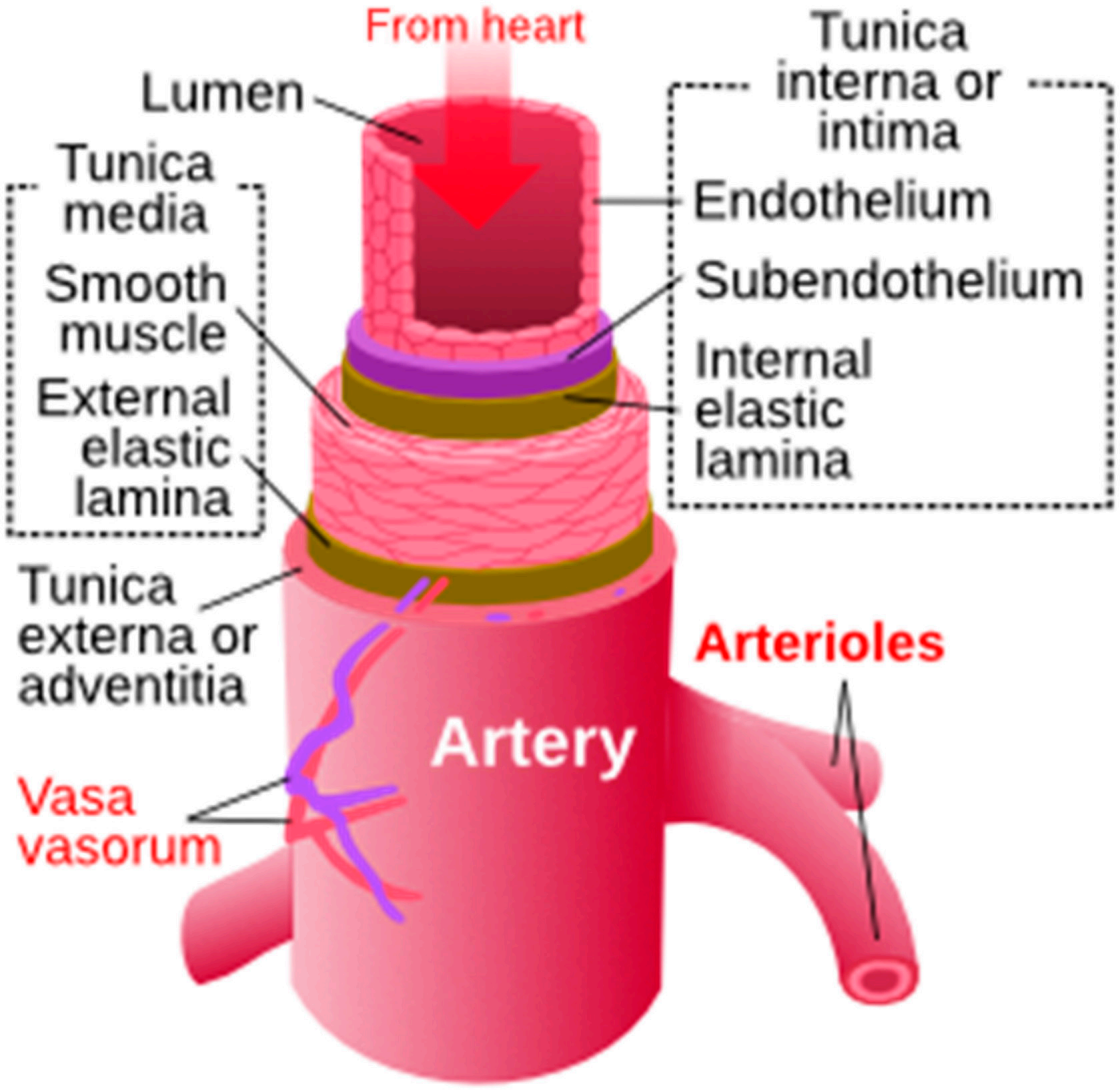
The structure of an artery that should be significantly recapitulated in a healed, integrated, truly biocompatible vascular graft (anatomical features of the illustration are not to scale). (Attribution—Kelvin Song, CC BY-SA 3.0 <https://creativecommons.org/licenses/by-sa/3.0>, via Wikimedia Commons.)

This article addresses the limited progress with vascular graft development. The article develops the hypothesis that the “biocompatible” biomaterials comprising synthetic grafts are, in fact, questionably biocompatible. The long-term “attack” by the body on vascular graft biomaterials diminishes performance and accelerates graft failure. To expand upon this hypothesis, when meaningful biocompatibility is achieved, we will have much improved synthetic vascular prostheses. Biocompatible, in the context of the hypothesis presented here, means recapitulating key functional elements of the anatomy of the blood vessel. The anatomy of a heathy blood vessel is illustrated schematically in Fig. 1.

We will examine here the historical roots of vascular grafts, focusing on observations important to the biocompatibility hypothesis. New developments and strategies will be presented including tissue engineering approaches. Concepts surrounding biocompatibility will be briefly addressed. Finally, this article will justify the biocompatibility hypothesis for vascular grafts.

Note that there are many review articles on vascular grafts. Just a few of these are cited here, and others are cited further into this article [1–5]. This article does not aim to be comprehensive in reviewing the vascular graft literature. It is focused on explaining why, after 80 years of development, we have seen little progress.

## Historical

Articles and books have been written discussing the historical roots of vascular grafts [6–9]. This section will focus on historical observations relevant to the biocompatibility hypothesis proposed here in this article.

The modern synthetic vascular prosthesis is often attributed to Dr Arthur Voorhees [10,11]. Vorhees observed what was then referred to as a “pseudoendothelial layer” on multifilament sutures implanted in the bloodstream. This coating, composed of compacted fibrin, was smooth, glistening, and relatively free of thrombotic material. He surmised that a fabric in the bloodstream might evolve such a coating and this surface would be desirable for the luminal surface of vascular grafts. After exploring a number of different fabrics in a dog model with limited success, he tried Vinyon-N, a poly(vinyl chloride) fabric (Union Carbon and Carbide Corporation), fabricated into a tube on his wife’s sewing machine. He demonstrated promising aortic grafts in dogs, reported in 1952 [11], and a first-in-human implantation in 1954 [10]. Soon thereafter, Dacron and nylon were explored for vascular prostheses. Woven Teflon was also explored in this period.

To illustrate the enthusiasm for such synthetic vascular grafts in the days before a medical device industry embraced vascular prostheses as a commercialized product, consider this quotation from a 1958 book on surgical materials [12].

“The Terylene, Orlon or nylon cloth is bought from a draper’s shop and cut with pinking shears to the required shape. It is then sewn with thread of similar material into a tube and sterilized by autoclaving before use.”

Nylon grafts failed rapidly due to degradation—the amide backbone chemistry of nylon is identical to the amide backbone of proteins and the body had excellent mechanisms for breaking down unwanted polyamides. Dacron (Terylene) weaves and knits dominated the field in the early days of vascular prostheses. A “trellis” concept was proposed by Dr Lester Sauvage to explain healing in vascular grafts with the goal to generate a living, functioning endothelial lining [13,14]. Sauvage subsequently wrote articles highlighting how endothelial healing, though noted in experimental animals like baboon and pig, was rarely seen in humans [14–16]. Stratton et al. [17] determined that aortic Dacron grafts, even a year after implantation in humans, continued to react with blood platelets.

Expanded Teflon, an exceptionally biostable material, was the next important advance in vascular grafts. ePTFE, fabricated by a process of extrusion, heating, and stretching, was patented by W.L. Gore Inc. in 1969. Numerous studies established that in many sites, ePTFE and Dacron performed similarly with respect to long-term patency in humans [18,19]. There have been some studies in the literature on heparin surface-modified vascular grafts. Overall, heparin modification has not led to increased vascular graft success [20].

Relevant to this article are specific, seminal studies in baboons by Dr Alex Clowes and colleagues [21]. They demonstrated that the pore size of the ePTFE used clinically (nominally 30 μm based on internodal distance) was too small to allow blood vessel ingrowth from the surrounding tissue. When they used 60-μm Gore-tex, they found rich blood vessel ingrowth and luminal endothelialization. They concluded that based on 3 possibilities for reendothelialization—anastomotic pannus ingrowth, deposition of endothelial precursors from the bloodstream, or ingrowth of vessels and endothelial cells through the walls of the graft—ingrowth was most likely the driver of luminal endothelialization. This ingrowth is somewhat analogous to larger natural blood vessels that have their own internal blood vessel network, the vasa vasorum. When Clowes and colleagues sought to bring this exciting observation of reendothelialization to the clinic, the U.S. Food and Drug Administration (FDA) had concerns over the burst strength of the grafts and insisted that for the studies to proceed, grafts with a sturdy external wrap be used. Unfortunately, that external wrap would not permit periluminal blood vessel ingrowth. In the clinical trials, the 30- and 60-μm Gore-tex grafts showed similar outcomes and so the hypothesis that ingrowth of blood vessels from exterior tissue to the graft would lead to endothelialization could not be supported.

**Fig. 2.**
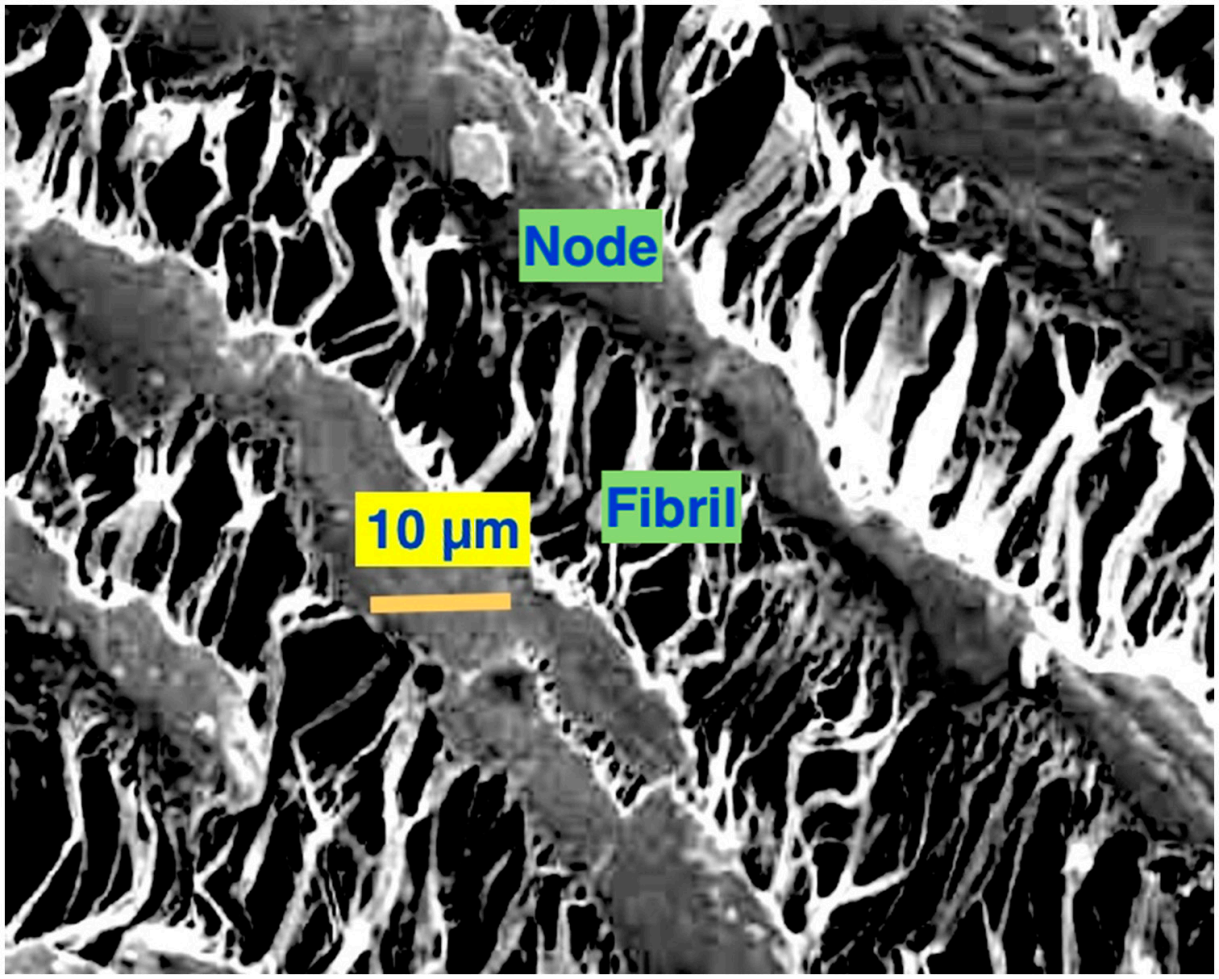
Scanning electron microscopy image of ePTFE showing the fibril–node porous structure.

Porosity is an important element in vascular prostheses. The 30- and 60-μm designations expressed above are related to the distance between the nodes (Fig. 2). The porous structure of ePTFE shown in Fig. 2 is clearly complex, and the “30 μm/60 μm” designations do not fully characterize the complex porosity of this material. For woven and knitted fabric grafts, the descriptions of porosity are also complex and involve an average pore size based on a distribution of pores and channels [22].

Polyurethanes are mechanically strong polymers that offer numerous fabrication possibilities and have often been used in medicine. The earliest use of polyurethanes for vascular grafts may be in a 1960 study led by cardiovascular device pioneers, Tetsuzo Akutsu and Willem Kolff [23]. They compared solid polyurethane tubes and foam polyurethane tubes in a dog implantation model. They reported improved performance (less thrombosis) for the foam tubes versus the solid tubes. They noted extensive fibrosis in all grafts. They expressed concern for the long-term biostability of the grafts. Since that time, hundreds of papers have been published on polyurethane vascular grafts. At least one graft type, Vectra (Bard Vascular Access), has been used in humans for dialysis vascular access. The published studies on Vectra in the literature (most around 2008) suggest similar long-term performance to ePTFE for dialysis arterio-venous (AV) graft vascular access, i.e., complication prone and subject to thrombotic failure [24,25]. The disconnect between the large number of studies on novel polyurethane vascular grafts (since 1960) and their failure to translate to significant clinical application suggests the fundamental advantages of polyurethanes (strength, elasticity, many options for fabrication, and surface modification) are tempered by troubling long-term outcomes, particularly thrombosis, hyperplasia, and biodegradation [26].

Grafts fabricated of natural tissue are another category of vascular prostheses that have had an impact on vessel replacement and are also relevant to central themes in this article. The earliest vascular replacements were harvested, preserved blood vessels (artery allografts) [8,9]. Although revolutionary to medicine at that time and demonstrating reasonable efficacy, there were issues in obtaining sufficient numbers of functional blood vessels, correctly sizing them, and longer-term durability. When Dacron grafts were introduced, these early tissue allografts were largely phased out. However, other approaches using biological tissue were, and still are, being explored. The Sparks mandril approach involved the implantation of an inert mandril (rod), harvesting the foreign body capsule around the rod (often with an embedded Dacron mesh) and implanting the tubular structure obtained [27]. Although the collagen tubes produced by this method often showed early success, longer-term results were mixed. Issues of thrombosis and graft degradation were noted. This mandril concept is still being considered some 60 years after its inception [28]. Vascular prostheses composed of fixed (crosslinked, often with glutaraldehyde) umbilical veins, collected in hospital delivery rooms, were pioneered by Herbert Dardik [29]. Some success has been noted in many clinical trials, but other reports of serious complication with this graft have been published [30]. To address immunogenicity, the Dardik graft is processed with crosslinking agents. Other groups are exploring decellularization to remove immune-reactive cellular components from harvested vessels [31–35]. The general approach of decellularizing blood vessels is used for creating larger-diameter vascular prostheses. Commercialized products include Artegraft (bovine carotid artery) [36,37], Solcograft (bovine carotid artery—possibly no longer available), ProCol (bovine mesenteric vein), and SynerGraft (bovine ureter) [31]. Artegraft has demonstrated good clinical outcomes for lower extremity bypass [38].

At this time (2022), the most widely used vascular graft materials for coronary artery repair are autogenous saphenous vein and internal mammary artery. Autologous vessels still outperform decellularized vessel grafts and are the optimal standard of care, although bovine carotid artery does perform acceptably [39].

Tissue engineering and tissue regeneration offer the possibility of creating living vascular graft structures that might emulate the vascular structure shown in Fig. 1. There are many review articles aimed at summarizing and commenting upon this growing literature [40–53]. An early tissue regeneration approach applied pig small intestinal submucosa (SIS) as vascular prostheses [54–57]. Although good success, often in comparison to ePTFE grafts, was noted in these papers published from 1989 to 2004, this SIS material as a vascular replacement is not mentioned in the recent literature, although many other applications for SIS are now in clinical practice. Two tissue-engineered vascular grafts have translated to clinical studies. Cytograft, based on cell sheet engineering, was implanted as an arteriovenous fistula for dialysis access for up to 9 months [58]. Good outcomes, including lack of immunological attack, were noted. Further clinical studies were performed with reasonable success. The process to create the Cytograft prosthesis was time consuming, and each graft might take 6 to 9 months to completion. The company has since closed its doors. Humacyte Inc. creates a vascular conduit by culturing smooth muscle cells (or, more recently, human induced pluripotent stem cells) on a biodegradable scaffold, mechanically stimulating the construct to increase production of extracellular matrix, and, finally, decellularizing the construct [32,41,59–61]. Clinical trials reported in 2022 documented 11 patients who had used the Humacyte graft for 60 months [62]. Results from the complete trial suggested a 58% patency rate for 10 of the patients. Another tissue engineering approach using fibroblast-produced extracellular matrix (donor dermal fibroblast remodeling of fibrin gels exhibiting healing and growth after recellularization) shows promise in large-animal models and early clinical investigations [63,64].

An approach for tissue engineering, referred to as in situ tissue engineering [65], has generated some interest due to satisfactory outcomes. This general concept, using the body as the bioreactor to generate new tissue, has been applied to vascular prostheses. One starts with a degradable, tubular, porous scaffold that might be inserted directly into the bloodstream. This concept is similar to the use of a porous fabric such as Dacron, except that the biomaterial scaffold ultimately degrades away and the new wall structure, starting with fibrin clot, is reorganized by cells such as the macrophage to a natural tissue, blood vessel-like structure. Many publications have appeared exploiting this concept, and importantly, outcomes (patency, endothelialization, etc.) are generally positive compared to control materials such as ePTFE [66–70].

The large numbers of studies, preclinical and clinical, for tissue-based vascular conduits highlight the interest in this approach, and there is some evidence that good outcomes can be achieved. However, the field has been plagued for 30+ years by concerns with thrombosis and aneurismal failure (loss of mechanical integrity), especially after long implantation. Also, issues of scale-up and high cost have been challenging for the field, particularly with tissue-engineered grafts. On the other hand, the basic “biocompatibility” of natural tissue and extracellular matrix proteins continues to suggest that these approaches have merit and are worth continued exploration and development.

## Summary of Key Points Leading to the Biocompatibility Hypothesis

After this brief review of historical and modern literature on approaches to replace blood vessels, especially small-diameter vessels, the following issues are highlighted. Central to this summary is: “How well do approaches to emulate or replace blood vessels recapitulate elements of native, living blood vessels?” The lack of a healed endothelial layer, particularly in humans (and older or diseased humans), is the less-than-desirable outcome most frequently observed. Also, smooth muscle cells and elastin should be present within the walls of properly healed blood vessels. The mechanical compliance of the replacement blood vessel should match that of the natural vessel to prevent flow disturbances [71] thought to exacerbate hyperplasia [72]. For synthetic polymeric vessel replacements, porosity is critical. Finally, the long-term durability of replacement vessels, particularly natural tissue, tissue-engineered vessels, and polyurethane vascular grafts, has been a significant issue. The challenges of modeling (i.e., predicting) degradation failure where it occurs after many years and the potentially catastrophic consequences of aneurismal failure cloud the literature on many vascular graft innovations. These points will be elaborated upon shortly.

## Issues to be Considered in Reviewing the Vascular Graft Literature

The vascular graft literature is extensive. Conclusions from one study may not be relevant for another study done under different conditions. In reviewing this literature, here are some considerations. The numerous experimental protocols (variables) that populate this vascular graft literature often make it challenging to intercompare studies and to arrive at justifiable conclusions.

### Graft segment length

In general, the longer the segment length, the more prone the graft is to thrombotic occlusion. In the literature, you will find segment lengths from 1 cm to segments as long as 25 cm. A consideration that is rarely addressed is: How does the segment length scale with the size of animal (mouse? human?) receiving the implant? A human cardiovascular bypass graft is typically 10 to 20 cm in length [73].

### Graft diameter

There are no FDA-approved small-diameter (≤4 mm) grafts—such grafts fail primarily due to thrombosis. Clinically, a 3- to 4-mm graft would be valuable for peripheral limb repair and a 2- to 3-mm-diameter graft would be applied for coronary artery repair. There are numerous studies showing acceptable results with 1-mm-diameter grafts in rats. But 1-mm-diameter grafts have never been introduced for human applications. This is another example of the issues in scaling devices between experimental animals and humans.

### Animal model

Numerous animal models have been explored with the objective of predicting vascular graft performance in humans [74–76]. None of these animal models perfectly emulates human hematology, although some, like the baboon, come close [77]. Also, consider that most cardiovascular disease occurs in older individuals, yet our animal models are mostly young animals. Particularly relevant for this article is that an animal model should emulate the slow vascular endothelium healing seen in older humans. Of all the common animal models, the slow endothelial healing observed in sheep parallels slow healing in humans [76].

### Implantation site

In clinical medicine, common implantation sites for vascular prostheses include upper aortic, femoropopliteal, aortoiliofemoral, and vascular access artery–venous sites in limbs. The blood flow rate and native vessel diameter in different implantation sites can be more, or less, challenging for long-term functionality of vascular prosthesis. In humans, most common vascular graft materials work acceptably in large-diameter (upper aortic) vessels.

### Anastomosis

Sutures at the distal and proximal anastomoses can be continuous or interrupted. Continuous sutures require less surgeon time. However, where newer graft materials are engineered to match the compliance of the native blood vessel, continuous sutures create a flow disturbance at the anastomoses that can lead to thrombus and hyperplasia. Interrupted sutures, though time consuming for the surgeon, greatly reduce flow disturbances at the interface of native vessel and graft polymer. Dr Lester Sauvage, a pioneer vascular surgeon and mentor to the author of this article, emphasized the quality of the suturing (tight, uniform, appropriately spaced) as being important to the success of vascular prostheses.

### Graft polymer composition

A tremendous number of compositions and chemistries have been described in the vascular graft literature. Also, many polymer surface modifications have been explored in the literature. Regulatory agency-approved graft materials are largely confined to Dacron and ePTFE. There are special considerations for vascular graft biomaterials: long-term durability, mechanical strength (burst strength), mechanical compliance, kink resistance, and suturability. Also, vascular graft materials must meet ISO10993 qualifications.

Clearly, there are also differences in the thrombogenicity of different biomaterials, although precise rules to “rank” thrombogenicity on a quantitative scale have not been defined [78–80]. The importance of graft material blood compatibility is, however, open to debate. Upon implantation, the patient or animal is anticoagulated, and that anticoagulation regime is continued for some days after implantation. Thus, thrombosis on even a thrombogenic biomaterial might be inhibited. After a few days, at the surface of the porous structure of a synthetic graft, a smooth, glistening luminal layer of compacted fibrin is noted (the observation that goes back to the early work of Dr Voorhees). This layer is micrometers thick, and the biomaterial of the graft is well overcoated by this layer. Grafts used in the 1970s to 1980s were, in fact, preclotted with the patient’s blood to seal the pores to prevent oozing blood, creating a micrometer-thick clot layer that would remodel in a day or two to the glistening compact fibrin layer. Because of systemic anticoagulation, early in the graft biointegration process, thrombosis is pharmacologically blocked. After anticoagulation is discontinued, the blood only sees the fibrin layer and never sees the graft polymer. This leads to the conclusion that blood compatibility may not be an important criterion for graft polymers and also suggests that later-stage thrombotic failure can be attributed to flow disturbances and low blood flow rate.

### Porosity

Early studies of vascular grafts concluded that a porous surface will outperform smooth, solid tubes [81]. Based on those early observations, most synthetic vascular graft materials described in the literature are porous. Porosities vary from nanoscale to micrometer scale. Most materials have a broad range of pore sizes, so an average pore size is frequently quoted. Figure 2 demonstrates with ePTFE the complexity in accurately defining porosity. A 1973 study demonstrated that by varying the porosity of an implant, the foreign body capsule and the macrophage response could be significantly modulated [82]. This point will be further elaborated upon in the section below discussing approaches to enhance the biocompatibility of implants.

### Duration of implantation

Most cardiovascular implants to be assessed in animal studies are implanted for about 1 month, a time period where the most active response seen in the first hours and days of implantation subsides to a long-term, low-level reaction. Sometimes, animal studies use implants for 3 or 6 months. In preclinical research, we rarely do longer studies because of costs, ethical issues, and considerations of the lifetime of the animal (for a mouse that might live 1 year, how does a 1-month study compare to a human that might live 75 years?). Since a vascular prosthesis is presumably implanted “forever,” the long-term outcome, so difficult to measure experimentally, is relevant. Degradation and aneurism, where observed, frequently occur in the 5- to 10-year implant period.

### Histology performed

The outcomes of vascular graft implantation, particularly in animals but sometimes in humans, are often measured with histological analysis of harvested tissues and devices [83]. Key elements to address in histology, to assess healing and integration, are the collagen capsule (Masson’s trichrome or Sirius red plus immunostains for specific collagens), elastin (Verhoeff stain), and immunostaining for endothelial cells (blood vessels), macrophages (M0, M1, M2), and smooth muscle cells. This extensive histological analysis will help to distinguish between the classic foreign body reaction (FBR) and a reconstructive healing (to be elaborated upon in the section on the biocompatibility hypothesis).

## The Biocompatibility Hypothesis

Based on the background information to this point, a hypothesis is presented here that could open pathways to explore this key question:

Why after 70+ years of graft development and thousands of high-quality published studies has there been no significant progress in the clinic, particularly where smaller-diameter vascular grafts are needed?

Biomaterials today are qualified as biocompatible by passing relevant ISO10993 tests [84,85]. Many of the ISO tests look at extracts from the biomaterial (soluble or leachable components) and then assess their effects on cells in culture or their effect injected systemically or locally in animals. All materials having zero or very low levels of leachable substances will receive similar ranking in these tests. Then, the materials would be subjected to specific tests for response in soft tissue or response in blood [86].

A widely quoted definition of the word “biocompatibility” was published based on discussions at a 1986 biomaterials consensus conference held in Chester, England and, more recently, at a consensus conference in Chengdu, China [87,88].

“the ability of a material to perform with an appropriate host response in a specific application”

This definition is accurate but does not suggest ways to measure biocompatibility or rank materials as more or less biocompatible, and it offers no insights on how to improve or enhance biocompatibility.

**Fig. 3.**
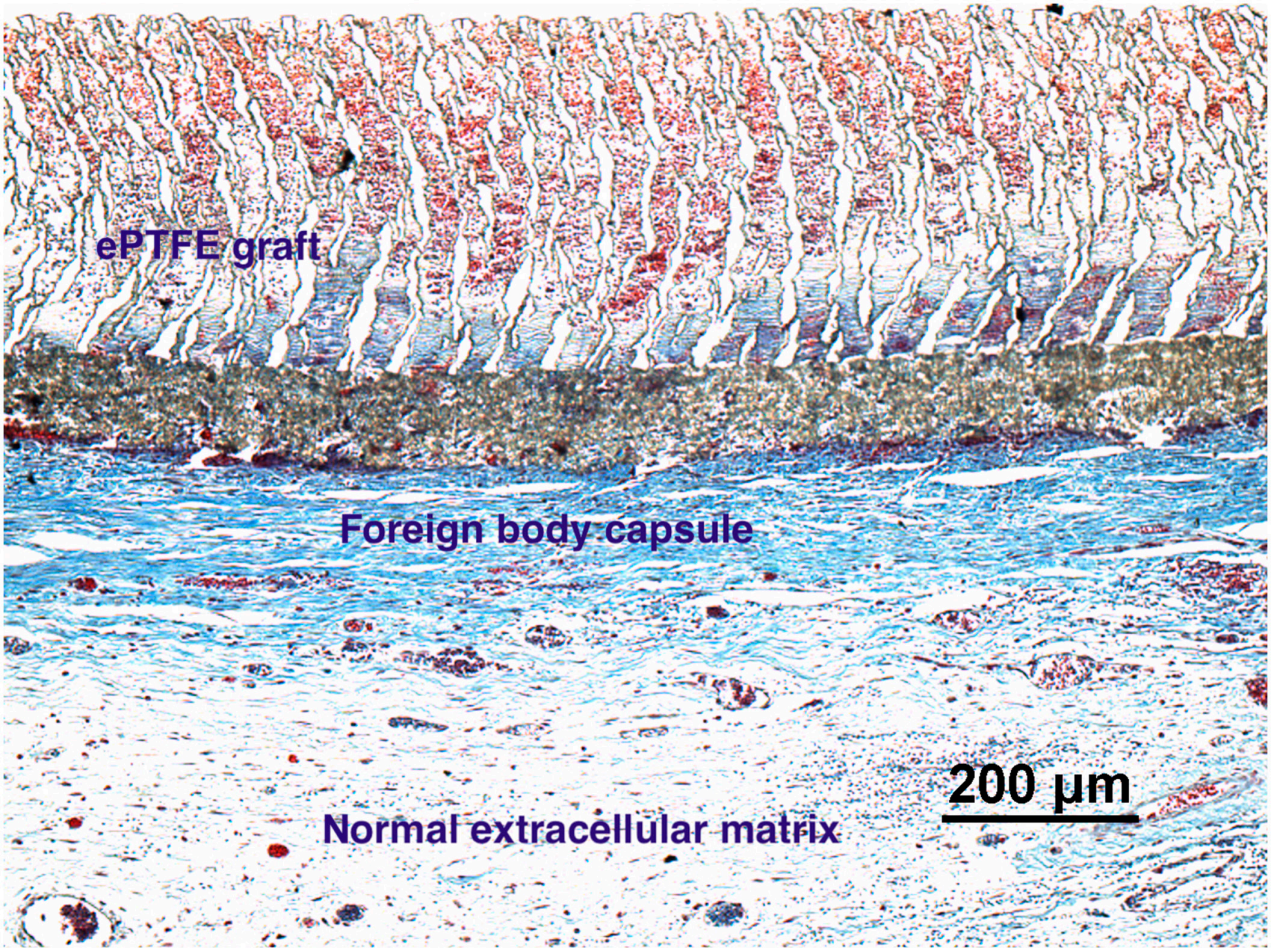
An ePTFE graft after implantation in a sheep for 1 month. Trichrome stain—the foreign body capsule (collagen) is stained darker blue. The central gray zone is a reinforcing wrap around the graft as fabricated by the manufacturer. (Data of Le Zhen, Louis Chen, Elina Quiroga, Jonathan Himmelfarb, and Buddy Ratner.)

**Fig. 4.**
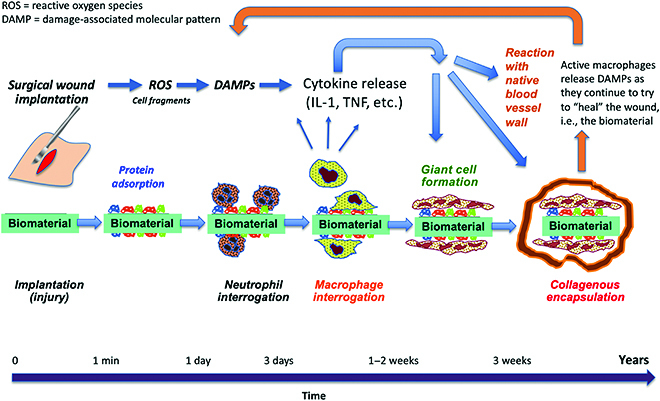
The time sequence of the inflammatory reaction to implanted “biocompatible” biomaterials. Since the biostable biomaterial (a foreign body) can never be digested by the macrophages, macrophages continue sending signals maintaining the encapsulated state. These same signals also negatively impact the functions of the blood vessel cells (hyperplasia, thrombosis).

Consider, for this discussion, the soft tissue response. For biomaterials that have passed the ISO leachables tests, when these materials are implanted in living, soft tissue, similar outcomes are noted. After about 3 weeks, the implant is surrounded by a dense, fibrous, collagenous capsule (typically 50 to 150 μm thick), the FBR. The capsule has low vascularity. Within and beneath the capsule, at the interface with the biomaterial, macrophages and giant cells are noted, even years after implantation. The presence of this fibrous capsule is well documented for vascular grafts [89]. Figure 3 shows the histology of an expanded Teflon (ePTFE) graft after 1 month in a sheep, particularly highlighting the fibrous capsule. Figure 4 schematically illustrates the time sequence of the reaction to implanted biocompatible biomaterials.

The biocompatibility hypothesis proposed here questions whether this well-accepted reaction is indeed biocompatible. The body isolates, rather than integrates, the biomaterial. The body would do this to a splinter or a bullet. Scar healing and the FBR have similarities—dense collagen and low vascularity. Scar healing is generally considered to be the opposite to regenerative or reconstructive healing. Also, all “biocompatible biomaterials” (with a few exceptions to be discussed shortly) give almost identical reactions. Are all biomaterials, and a splinter, and a bullet equally biocompatible? The biocompatibility hypothesis presented here proposes that truly biocompatible biomaterials will heal into vascularized, reconstructed tissue sites, recapitulating the original tissue into which they have been implanted (i.e., no dense scar).

For vascular grafts, this hypothesis has significant implications beyond those for many other implants. The FBR capsule is constrictive, a phenomenon also noted with breast implants. This contraction around the vascular graft tubular structure can reduce the diameter of the vessel, creating flow disturbances and lowering blood flow rate. This contraction potentially leads to increased thrombosis. Also, the capsule is rigid (nonelastic). When a pulse of blood from the arterial system hits the nonelastic vascular graft (ePTFE, Dacron, the rigid fibrous capsule), a flow disturbance is created, which might exacerbate hyperplasic cell proliferation [90], a major cause of graft failure. Also, the avascular nature of the capsule and the low vascularity within the porous structure of most synthetic grafts may inhibit endothelial healing [21]. Finally, the macrophage secretion of proinflammatory cytokines [for example, interleukin-1β (IL-1β) and tumor necrosis factor-α (TNF-α)] by the graft material over time [91] may be inhibitory to healing of grafts. In addition to these proinflammatory molecules, activated macrophages secrete numerous potent oxidizing agents and enzymes [92] and these probably contribute to the degradation seen so commonly in natural tissue grafts and in polyurethanes (which vaguely resemble the amide backbone structure of proteins).

In the 1980s, the writings and research of James M. Anderson alerted the biomaterials community to the significance of the macrophage in understanding the FBR [93,94]. Two more contemporary concepts have further clarified the reactions occurring at the biomaterial surface upon implantation. These are the understanding of macrophage polarization (the “M1 M2” concept) and of damage-associated molecular patterns (DAMPs).

The M1 M2 concept was clarified and well described by Mantovani and colleagues [95,96]. The concept expands upon the long-standing observations of macrophage heterogeneity in the appearance of the macrophage and its behavior. Broadly, molecules such as IL-1, TNF, lipopolysaccharides, and reactive oxygen species (ROS) stimulate the monocyte or macrophage to an M1 polarization, a highly active form of the macrophage associated with injury and attack of pathogens. An M2 polarization of the macrophage, stimulated by signaling molecules such as IL-4, is associated with reparative or regenerative healing. In a simple model, upon injury, the molecules released from damaged cells and invading pathogens trigger the monocyte (or macrophage) to an M1 form to clean up the injury site of damaged tissue and bacteria. When that initial, active M1 phase is complete, the macrophage response transitions to M2 to heal or repair the damage. It is well recognized that the M1–M2 designation does not clearly define 2 distinct subsets of macrophages but represents a continuum of states from M1 to M2. The M1 M2 concept has been applied to vascular grafts to clarify improved healing of a particular graft [66].

The DAMP concept stems from earlier immunology ideas defining pathogen-associated molecular patterns (PAMPs), a mechanism to recognize microbes and launch an attack on them [97]. DAMPs bring these molecular protection ideas to nonpathogen damage mechanisms, i.e., tissue injury. Because DAMPs are not associated with pathogens, the term “sterile inflammation” is sometimes used [98]. DAMPs directly activate the innate immune system, but also turn on the adaptive response. A surgical implantation of a biomaterial is always associated with tissue damage. ROS species formed in the wound were early on recognized as a trigger for DAMPs [97]. Some DAMP molecules relevant to this article are fibrinogen, IL-1α, and a number of extracellular matrix components (Wikipedia lists 34 different categories of DAMP molecules).

The biocompatibility concept proposed in this perspective article starts with the premise that all “biocompatible biomaterials” that are declared “biocompatible” by ISO standard tests are, in fact, not all that biocompatible. These ISO tests include ISO10993-5 for cytotoxicity and 10993-6 for local effects after implantation. Important to the hypothesis proposed here, 10993-6 considers an uncomplicated, thin foreign body capsule as meeting the “biocompatibility” acceptance criteria. The fact that the body tries to wall off or isolate the biomaterial, rather than integrating or healing it, is a central observation supporting this contrarian idea on biocompatibility. Relevant to this hypothesis and to this paper, perhaps the less-than-ideal performance of vascular prostheses can be attributed to the fact that the body is attacking the prosthesis, rather than integrating it into the normal biology? By “attacking,” this is to suggest that macrophages are polarized to the M1 phenotype and that DAMPs are released. Normally, macrophages perform their physiologic function and then disappear from the wound site. When long-term macrophages are present in a nonhealing wound, this would be classified as chronic inflammation [99]. The macrophage cannot remove (engulf, digest) the offending foreign object (the biostable, biocompatible biomaterial). Macrophages (and giant cells) are found at the biomaterial interface even years after implantation. Perhaps these macrophages are still releasing DAMPs? These DAMPs may react with cells in blood vessel walls leading to hyperplasia [100] and may inhibit endothelial cell proliferation.

The hypothesis presented and the scenarios described help to explain the concerns with the long-term performance of vascular prostheses. If they are recognized by macrophages as foreign to the normal physiology, the long-term biological attack will lead to aberrant healing and/or degradation. In the case of Dacron or ePTFE large-diameter vascular grafts, blood flow rates are so high that thrombotic occlusion or low degrees of hyperplastic narrowing are not important and so they function adequately. On the other hand, the macrophage attack on natural tissue vessels, large diameter or small, can lead to structural breakdown with the potential for aneurism and rupture.

Based on this hypothesis—that today’s vascular grafts are continually under attack by the body because they are not biocompatible—what prospects are there to address these issues for the development of future vascular prostheses that can function well in small-diameter sites? Strategies to develop grafts that truly heal and integrate are reviewed in the next section of this article.

## Strategies to Enhance Biocompatibility

At first glance, it might seem we are constrained to the classic FBR for our implanted biomaterials. However, there is evidence that synthetic biomaterials and natural tissue biomaterials that heal without the FBR can be developed. Many such materials have been summarized in a review article [101]. Three of these strategies that are particularly applicable to vascular grafts are elaborated upon here: porous materials with uniform 30- to 40-μm pores, decellularized natural tissue, and super-nonfouling biomaterials.

Biomaterials where all pores are spherical, 30 to 40 μm in diameter, and interconnected were first described in the literature in 2004 [102]. These porous structures, prepared by sphere templating, were found to heal subcutaneously with little fibrosis and robust, interpenetrating vascularity. Analogous porous structures but with pore sizes of 20 or 80 μm and larger did not show the impressive and unexpected biointegration seen with 30- to 40-μm pores. Subsequent studies in heart muscle and again subcutaneously confirmed low fibrosis, good vascularity, and the presence of large numbers of macrophages in the M2 polarization [103,104]. The composition of the structural material seems unimportant as long as it has the 30- to 40-μm interconnected pore structure and is deemed acceptable by the ISO10993 tests—such scaffolds have been fabricated from many hydrogel compositions, fibrin, silicone rubber, and polyurethane.

**Fig. 5.**
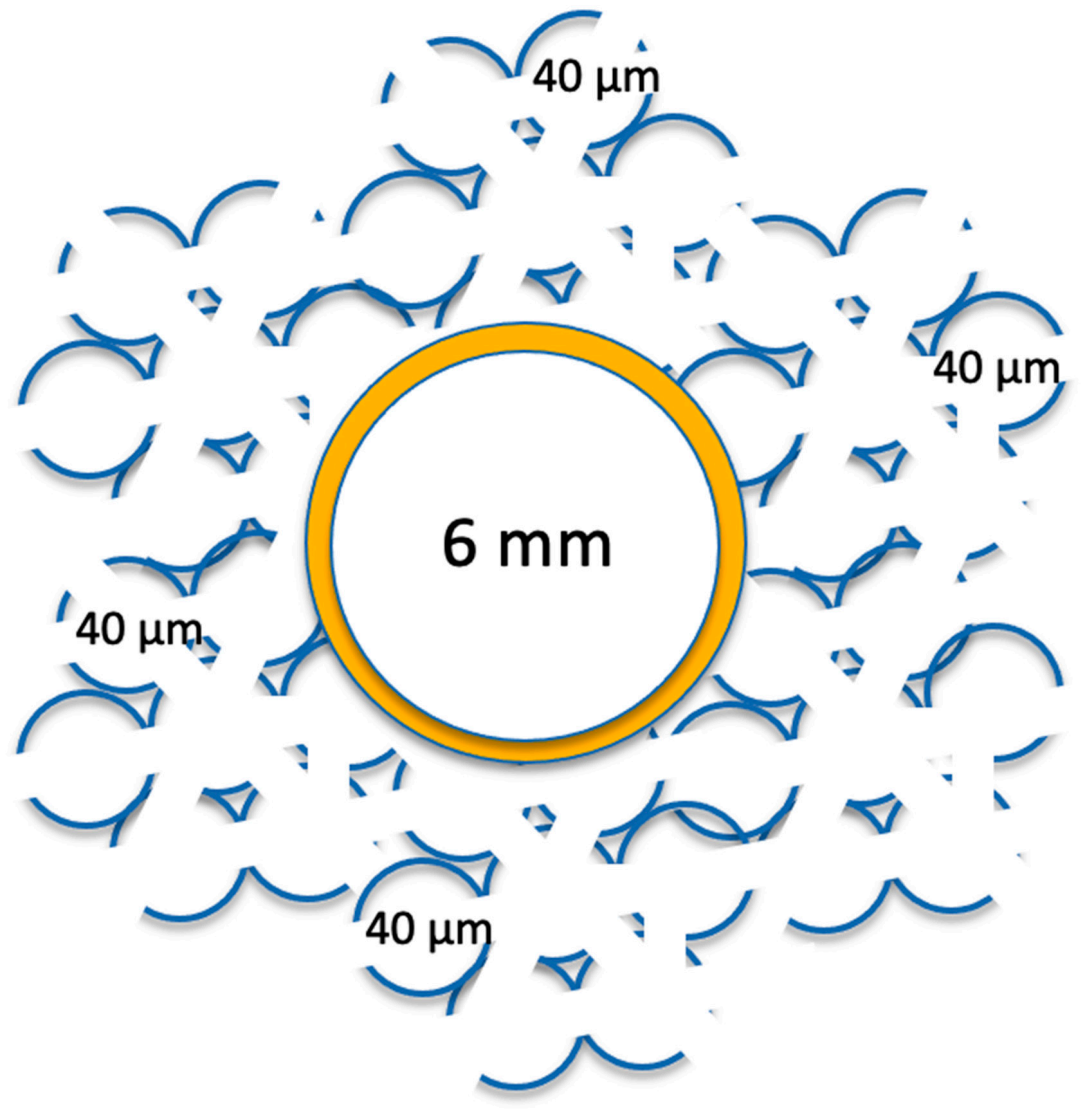
Schematic diagram of the wall structure of a polyurethane vascular prosthesis now under development at the University of Washington (inner and outer components are not to scale). The orange color suggests the thin gelatin layer lining the lumen of the graft.

This prohealing strategy using precision-controlled pore sizes of 30 to 40 μm has been applied to vascular prostheses. Healionics Corporation (Seattle, WA) has developed a vascular graft, now in clinical trials for dialysis vascular access, based on this idea. They start with a conventional, 6-mm ePTFE vascular access graft. To the outer surface of this graft, using a silicone adhesive, they adhere silicone elastomer granules prepared by grinding up 35-μm pore-size, sphere-templated silicone elastomer. Upon implantation in sheep (bilateral arteriovenous shunt mode) up to 12 weeks, they note significantly reduced fibrosis and reduced restenosis compared to ePTFE controls [105]. This 30- to 40-μm pore concept is being further explored in a vascular graft now under development at the University of Washington. The cross-sectional structure of this graft is illustrated schematically in Fig. 5. The graft permits transluminal ingrowth of blood vessels facilitating endothelial development on the inner lumen. It is composed of a new biostable polyurethane developed for this purpose and is mechanically tunable to achieve compliances similar to artery wall [106]. The lumen is lined with gelatin to prevent blood oozing and provide a smooth surface for initial blood flow. The gelatin layer degrades away within the first few days of implantation. Preliminary sheep implantations (to be published) show excellent luminal endothelial development compared to the ePTFE controls. At harvest, the new graft was observed to be pulsatile and flexible, while the ePTFE control appeared rigid without visible pulsation.

Another type of material that has been shown to heal in a reconstructive, regenerative manner is based on decellularized natural tissues [107,108]. As part of the reconstructive process seen with such decellularized materials, macrophages are driven to the M2 polarization [109]. The regenerative mechanism is thought to be associated with breakdown of the natural material to bioactive peptides that are then cleared by the body as long as long-term residence of the implant material in the implant site is not observed [110]. Excessive crosslinking and processing, though improving mechanical properties, may inhibit this early-stage regenerative mechanism by slowing breakdown. This may explain long-term issues with some tissue-based vascular prostheses. The inhibition of breakdown might lead to long-term immunological attack on the material, similar to the FBR, where macrophage proteolytic enzymes might weaken tissue prosthesis.

The third possibility to achieve nonfibrotic, reconstructive healing involves super-nonfouling surfaces. Poly(carboxybetaine methacrylate), a zwitterionic polymer, has been shown to be unusually effective in preventing protein and cell pickup, even in the presence of undiluted blood plasma [111]. In a 3-month subcutaneous implantation experiment, no foreign body capsule formation was noted and more macrophages were driven to the M2 phenotype compared to a control materials of poly(2-hydroxyethyl methacrylate) hydrogel [112]. Such a coating on a porous vascular prosthesis might inhibit the foreign body capsule and encourage a more regenerative healing

## Rethinking Biocompatibility

The definition we most often use for the word “biocompatibility” was coined in 1986, in the earliest days of molecular biology [87]. The concepts of cell surface receptor, cytokine signaling, the inflammasome, and other ideas that help us understand the FBR were not invoked when that definition was developed. With new knowledge of biointeractions from contemporary molecular biology and the new tools to analyze the in vivo reaction to synthetic materials, we can rethink biocompatibility. Living systems separate “biocompatible” biomaterials from the body with a dense, nonvascularized wall, and this suggests that these materials are not biocompatible. I refer to most of the materials we use today in medicine as “biotolerated”—they work acceptably in their intended application, but they will be encapsulated. The hypothesis expressed here will require testing to assess its utility. However, it suggests a new way to think about biomaterials, particularly the materials used to construct vascular prostheses. Perhaps truly biocompatible biomaterials in vascular prostheses will permit a functional endothelial lining that will lead to long-term successful outcomes, especially for smaller-diameter vascular procedures?

## Vascular Grafts: Technology Success/Technology Failure

After reviewing papers on vascular reconstructive surgery from the late 1940s and early 1950s, it is clear that huge progress has been made over the past 70+ years in developing reliable vascular grafts for many procedures. Their widespread use in medicine and their good outcomes for some procedures are the “success” of the technology that I refer to in the title of this perspective article. The “failure” is our inability to create smaller-diameter prostheses with reliable, long-term outcomes. Hopefully, some of the ideas presented here and the critical assessment of our present technology will lead to new developments in vascular prostheses that will extend our successes in vascular reconstructive procedures.
